# Beneficial Effects of Combined Use of Extracorporeal Membrane Oxygenation and Hypothermic Machine Perfusion in Porcine Donors after Cardiac Death for Liver Transplantation

**DOI:** 10.3390/jcm12186031

**Published:** 2023-09-18

**Authors:** Hiroyoshi Iwata, Hiromichi Obara, Tetsuya Nakajo, Hiroki Kaneko, Yuga Okazawa, Nur Khatijah Mohd Zin, Hiroki Bochimoto, Makito Ohashi, Yoko Kawada, Mizuho Ohara, Hideki Yokoo, Naoto Matsuno

**Affiliations:** 1 Department of Transplantation Technology and Therapeutic Development, Asahikawa Medical University, 2-1-1-1 Midorigaoka Higashi, Asahikawa 078-8510, Japan; a090058@al.asahikawa-med.ac.jp (H.I.); emonyu36@gmail.com (T.N.); hiroki05.knks@gmail.com (H.K.); mizuhoo@asahikawa-med.ac.jp (M.O.); 2 Department of Hepato-Biliary-Pancreatic and Transplantation Surgery, Asahikawa Medical University, 2-1-1-1 Midorigaoka Higashi, Asahikawa 078-8510, Japan; hidekiyokoo@asahikawa-med.ac.jp; 3 Department of Mechanical System Engineering, Tokyo Metropolitan University, 1-1 Minamiosawa, Hachioji 192-0397, Japan; obara@tmu.ac.jp (H.O.); okazawa-yuga@ed.tmu.ac.jp (Y.O.); 4 Department of Cell Physiology, The Jikei University School of Medicine, 3-25-8 Nishi-Shimbashi, Minato-ku 105-8471, Japan; khatijah.zin@gmail.com (N.K.M.Z.); botimoto@jikei.ac.jp (H.B.); 5 Department of Clinical Engineering, National Center for Child Health and Development, 2-10-1 Okura, Setagaya-ku 157-8535, Japan; ohashi-mk@ncchd.go.jp (M.O.); kawada-y@ncchd.go.jp (Y.K.)

**Keywords:** donation after cardiac death, extracorporeal membrane oxygenation, hypothermic machine perfusion, liver, scanning electron microscopy

## Abstract

Grafts from donors after cardiac death (DCD) have greatly contributed to expanding the donor organ pool. This study aimed to determine the benefits of subnormothermic extracorporeal membrane oxygenation (ECMO) and hypothermic machine perfusion (HMP) in a porcine model of DCD liver. Female domestic crossbred Large Yorkshire and Landrace pigs weighing approximately 20 kg were used. The abdominal aorta and inferior vena cava were cannulated and connected to an ECMO circuit for in situ perfusion of the abdominal organs at 22 °C for 60 min, 45 min after cardiac death. The pigs were divided into the cold storage (CS) group (*n* = 3), where liver grafts were preserved at 4 °C, and the HMP group (*n* = 3), where liver grafts were preserved by HMP at 8–10 °C. After 4 h of preservation, liver function was evaluated using an isolated liver reperfusion model for 2 h. Although the difference was insignificant, the liver effluent enzyme levels in the HMP group were lower than those in the CS group. Furthermore, morphological findings showed fewer injured hepatocytes in the HMP group than in the CS group. The combined use of in situ subnormothermic ECMO and HMP was beneficial for the functional improvement of DCD liver grafts.

## 1. Introduction

The number of patients waiting for liver transplantation is increasing. Furthermore, a shortage of donors has become a serious problem. The use of grafts from donors after cardiac death (DCD) greatly contributes to expanding the donor organ pool but is associated with a higher risk of primary nonfunction and ischemic cholangiopathy [1,2,3]. Preservation using machine perfusion (MP) has been reported to be more useful and beneficial for liver grafts obtained from DCD than cold storage (CS) [4,5,6,7,8]. Using MP, oxygen, electrolytes, and nutritional elements are continuously provided, and harmful metabolites are removed. Therefore, MP can help maintain, recover, and evaluate graft functions.

To further improve the outcomes of organs obtained from DCD, abdominal normothermic regional perfusion (RP) has been introduced as a method for reconditioning organs in the donor before retrieval [9]. In 2007, the Barcelona group described the first 10 human liver transplant recipients who received a liver from DCD procured according to the normothermic extracorporeal membrane oxygenation (ECMO) protocol [10]. Subsequently, normothermic RP has been proposed to restore blood flow (using ECMO technology) before organ recovery in DCD donors, which is more beneficial than conventional rapid recovery techniques to counteract ischemic damage and improve the recipients’ outcomes [11,12]. In recent years, the combined use of normothermic RP and MP has been suggested to improve transplantation outcomes of DCD. Therefore, these technologies are being increasingly used [6,13]. However, whether using RP or MP, normothermic perfusion requires complete metabolic support. A temperature of 22–25 °C allows minimal metabolic activity and has preventive effects on cell lysis [14]. This study aimed to determine the benefits of subnormothermic RP using ECMO and hypothermic MP (HMP) for livers from DCD using a large animal model.

## 2. Materials and Methods

### 2.1. Animals and Procedures

Two- to three-month-old female domestic crossbred Large Yorkshire and Landrace pigs weighing approximately 20 kg (Zen-noh Premium Pig; Zen-Noh, Ibaraki, Japan) were purchased from Tokyo Laboratory Animals Science (Tokyo, Japan). After securing arterial lines for measurement and venous lines for intravenous infusion in either the right or left external jugular vein, the excision procedures were performed under full anesthesia. Anesthesia was induced with 0.05 mg/kg of medetomidine hydrochloride (Dorbene vet; Kyoritsu Seiyaku, Tokyo, Japan), 0.25 mg/kg of butorphanol tartrate (Vetorphale; Meiji Animal Health, Kumamoto, Japan), and 0.25 mg/kg of midazolam (Dorumicum Injection; Maruishi Pharmaceutical, Osaka, Japan) and was maintained with 2% isoflurane (Forane VR; Abbot Japan, Tokyo, Japan). A midline laparotomy was performed, involving partial resection of the xiphoid process. To prepare for liver exposure, the hepatic artery (HA), portal vein (PV), and upper and lower parts of the hepatic inferior vena cava were dissected and taped. Although the bile duct was also dissected and severed at this point, a nutrient catheter was inserted into the common bile duct, and its distal end was ligated. Simultaneously, the right external iliac artery was isolated, and approximately 600 mL of autologous whole blood was obtained. Subsequently, cardiac arrest was induced with an intravenous injection of 2 mEq/kg of potassium chloride (KCl drip injection 15%; Maruishi Pharmaceutical, Osaka, Japan); this point was determined as the starting point of warm ischemia. Next, 3000 units (U) of heparin (Heparin Sodium Injection “AY”; AY Pharmaceutical, Tokyo, Japan) were intravenously administered before cardiac arrest. After 45 min, the liver was perfused with subnormothermic RP using ECMO for 60 min. At the end of the ECMO, the liver was rapidly removed while maintaining cold conditions with crushed ice. A standard bench preparation procedure was performed. In addition, the portal and arterial systems were cannulated using our original plastic perfusion cannulas of appropriate size. The isolated liver was perfused with 1000 mL of Euro-Collins solution (Euro-Collins solution “KCC”; Kyowa CritiCare, Atsugi, Japan) at 4 °C while being periodically inverted to ensure uniform perfusion throughout. After CS at 4 °C or HMP at 8–10 °C for 4 h, the liver graft was evaluated for 2 h using an isolated liver reperfusion (ILR) model. All animal experiments were conducted in accordance with the Guide for the Care and Use of Laboratory Animals at the National Center for Child Health and Development and approved by the National Center for Child Health and Development Animal Research Committee (protocol code A2021-001-co1 and approved on 1 April 2021).

### 2.2. Experimental Design

**Figure 1:**
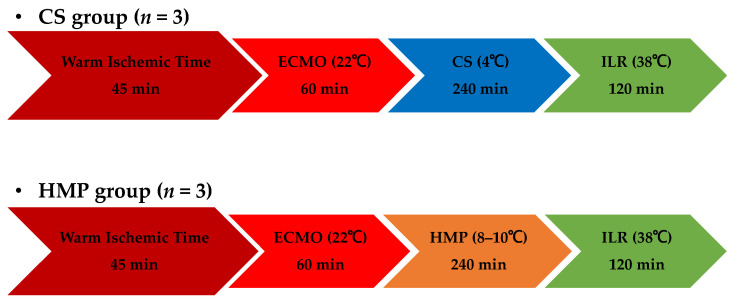
Experimental design. CS, cold storage; ECMO, extracorporeal membrane oxygenation; HMP, hypothermic machine perfusion; ILR, isolated liver reperfusion.

The two experimental groups were designated as follows: CS group (*n* = 3), in which liver grafts were preserved at 4 °C, and HMP group (*n* = 3), in which liver grafts were preserved at 8–10 °C (Figure 1).

### 2.3. Extracorporeal Membrane Oxygenation Protocol

The ECMO system comprised a centrifugal pump console (HCS-CFP; MERA, Kasukabe, Japan), centrifugal blood pump (HCS-MP23H; MERA, Kasukabe, Japan), extracorporeal circuit (Exceline Circuit N2; MERA, Kasukabe, Japan), and oxygenator (HPO-06H-CP; MERA, Kasukabe, Japan). After cannulation of the abdominal aorta and inferior vena cava, 3000 U of heparin was administered, followed by initiation of ECMO which ran for 60 min with the pump setting at 1.2 L/min. Blood pressure and temperature were set at 200 mmHg and 22 °C, respectively.

### 2.4. Preservation Protocol

**Figure 2:**
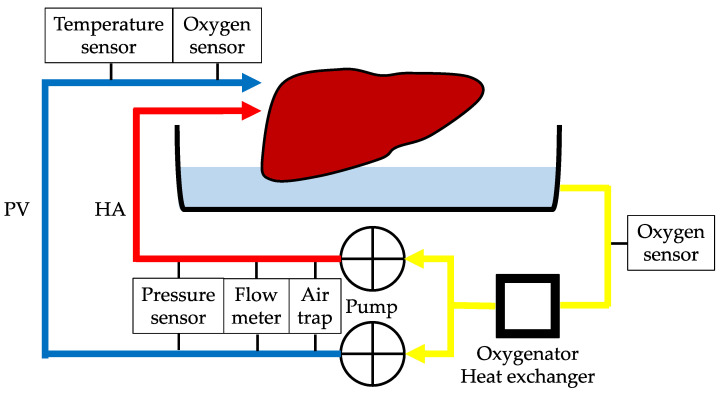
Machine perfusion system. HA, hepatic artery; PV, portal vein.

The preservation solution was a histidine-tryptophan-ketoglutarate (HTK)-based solution (Table 1). Regarding CS, the liver grafts were stored at 4 °C for 4 h. MP systems (Figure 2) consisted of HA and PV perfusion circuits. Each circuit comprised a roller-type pump (Masterflex 7520-40; Cole-Parmer, Bunker Court, IL, USA), electrical flow meter (VN05; Aichi Tokei, Aichi, Japan for PV; FD-SS02; Keyence, Osaka, Japan for HA), ceramic capacitive pressure sensor (KL76; Nagano Keiki, Nagano, Japan), and air trap developed in-house. An oxygenator (HP0-06 H-C; Senko Medical Instrument, Tokyo, Japan) was installed in the circuit for the HA and PV. The flow rate was set at 30–40 mL/min for HA and 120–150 mL/min for PV. The temperature of the preservation solution was maintained at 8–10 °C using a heat exchanger. The perfusion time was set at 4 h.

### 2.5. Isolated Liver Reperfusion

The ILR model was the same as an MP system. After preservation, all liver grafts were rinsed with 500 mL of cold Euro-Collins solution and subsequently exposed at room temperature (20–25 °C) without perfusion to simulate the slow rewarming of grafts during surgical implantation in vivo. PV and HA reperfusion was set at 5–8 mmHg and 80 mmHg, respectively, which were automatically maintained by a roller pump connected to a pressure sensor placed in the inflow line immediately before the arterial cannula. Autologous whole blood containing 30 mL of 8.5% calcium gluconate hydrate (Calcicol; Nichi-Iko Pharmaceutica, Toyama, Japan) and heparin (Heparin Sodium Injection “AY”; AY Pharmaceutical, Tokyo, Japan) was used as the reperfusion fluid. The livers were subsequently reperfused with oxygenated diluted autologous blood at 38 °C for 2 h. The hematocrit was maintained within the range of 10–12%. The oxygenator was regulated to achieve physiological blood gas values (partial pressure of oxygen, 150–200 mmHg; partial pressure of carbon dioxide, 30–50 mmHg) for ILR using a blood gas analyzer (ABL800 FLEX; Radiometer, Tokyo, Japan).

### 2.6. Primary Outcomes—Viability Assessment

To assess tissue damage, the levels of aspartate aminotransferase (AST), alanine aminotransferase (ALT), lactate dehydrogenase (LDH), and alkaline phosphatase (ALP) in the perfusate were measured every 60 min for ILR. Additionally, we performed a blood gas test for monitoring pH and lactate levels every 60 min for ILR using the iStat program (Abbott, Chicago, NJ, USA). The flow rate as well as resistance of PV and HA were monitored during the ILR.

### 2.7. Secondary Outcomes—Morphological Assessment

Liver tissue samples were collected for morphological evaluation after reperfusion. Samples were fixed in 10% phosphate-buffered formalin and dehydrated using ethanol. Paraffin-embedded sections (4 μm) were stained with hematoxylin and eosin and histopathologically evaluated.

The remaining samples were cut into small pieces and fixed using a fixative mixture of 0.5% glutaraldehyde and 0.5% paraformaldehyde in 0.1 M phosphate buffer (PB; pH 7.4) for 15–30 min at 4 °C. After fixation, the tissue samples were further fixed with 1% osmium tetroxide (OsO_4_) in 0.1 M PB for 8 h at 4 °C. These OsO_4_-fixed samples were washed thoroughly with 0.1 M PB and immersed in 25% dimethyl sulfoxide and then 50% dimethyl sulfoxide for 30 min each. The samples were then frozen on an aluminum plate chilled with liquid nitrogen and fractured into pieces using a screwdriver and hammer. After washing with 0.1 M PB, the samples were immersed in 0.1% OsO_4_ in 0.1 M PB for 96 h at 20 °C under light, followed by further fixation with 1% OsO_4_ in 0.1 M PB for 1 h at 4 °C. The samples were washed with 0.1 M PB and immersed in 1% tannic acid in 0.1 M PB, followed by 1% OsO_4_ in 0.1 M PB for conductive staining. The samples were dehydrated and lyophilized in a freeze dryer (VFD-21S; Vacuum Device, Mito, Japan) using t-butyl alcohol. The dried specimens were mounted onto a metal plate and coated with OsO_4_ in an osmium-coater chamber (HPC-1SW; Vacuum Device). After all the processes were completed, the specimens were evaluated using a field-emission scanning electron microscope (SEM) (Regulus 8100; Hitachi High Technologies, Tokyo, Japan) and observed at magnifications of 5000× (low magnification) and 20,000× (high magnification). Then, 10–15 images were captured for each specimen.

### 2.8. Statistical Analyses

The results are presented as mean ± standard deviation. Biochemical and hemodynamic data were analyzed using the Mann–Whitney U test. All statistical analyses were performed using EZR version 1.55 (Saitama Medical Center, Jichi Medical University, Saitama, Japan) [15], based on R and R commanders. Statistical significance was set at *p* < 0.05.

## 3. Results

### 3.1. Laboratory Test Results

**Figure 3:**
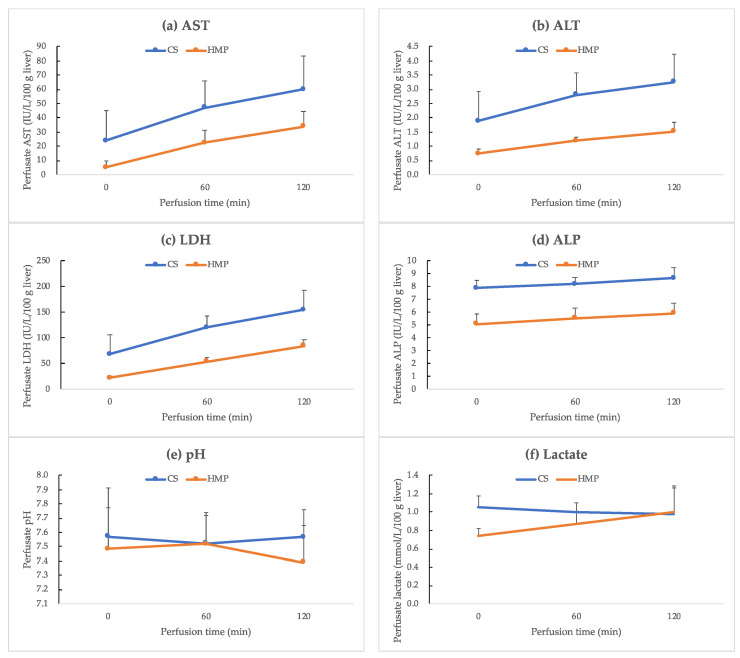
Laboratory test results. (**a**) Aspartate aminotransferase (AST) (IU/L/100 g liver), (**b**) alanine aminotransferase (ALT) (IU/L/100 g liver), (**c**) lactate dehydrogenase (LDH) (IU/L/100 g liver), (**d**) alkaline phosphatase (ALP) (IU/L/100 g liver), (**e**) pH, and (**f**) lactate (mmol/L/100 g liver) levels (mean ± standard deviation) in the perfusate during isolated liver reperfusion. Although the difference is not significant, the levels of AST, ALT, LDH, and ALP in the HMP group (*n* = 3) are lower than those in the CS group (*n* = 3). The pH and lactate levels did not differ between the groups. Statistical significance was set at *p* < 0.05. CS, cold storage; HMP, hypothermic machine perfusion.

Although the difference was not significant, the levels of AST, ALT, LDH, and ALP in the HMP group were lower than those in the CS group during ILR. At the end of ILR, the levels of AST, ALT, LDH, and ALP in the HMP and CS groups were 34.0 ± 10.7 and 60.1 ± 23.3 IU/L/100 g liver (*p* = 0.2); 1.5 ± 0.3 and 3.3 ± 1.0 IU/L/100 g liver (*p* = 0.1); 84.4 ± 11.2 and 154.9 ± 37.4 IU/L/100 g liver (*p* = 0.1); and 5.9 ± 0.8 and 8.7 ± 0.8 IU/L/100 g liver (*p* = 0.1), respectively (Figure 3a–d). The reference intervals of AST, ALT, LDH, and ALP for specific-pathogen-free 1-month-old crossbred Yorkshire and Landrace pigs were 4.6–17.5, 4.5–16.9, 63.2–137.0, and 40.9–143.1 IU/L/100 g, respectively [16,17]. The pH and lactate levels during ILR did not differ between the groups. At the end of ILR, the pH and lactate levels in the HMP and CS groups were 7.391 ± 0.257 and 7.569 ± 0.190 (*p* = 0.4), and 1.00 ± 0.29 and 0.98 ± 0.29 mmol/L/100 g liver (*p* = 0.7), respectively (Figure 3e,f).

### 3.2. Hemodynamic Results

**Figure 4:**
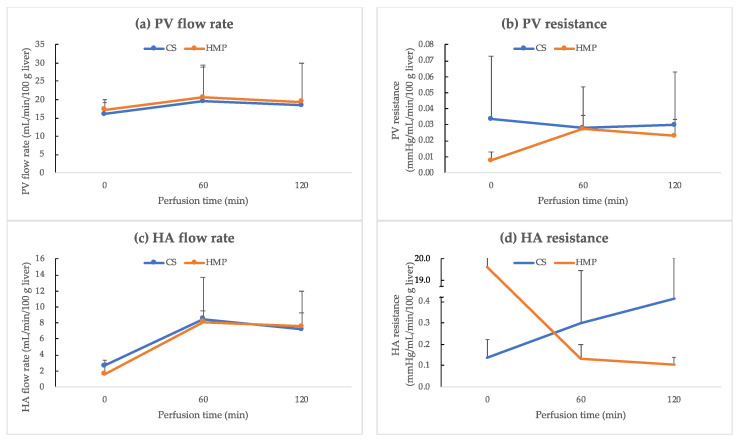
Hemodynamic results. (**a**) Portal vein (PV) flow rate (mL/min/100 g liver), (**b**) PV resistance (mmHg/min/100 g liver), (**c**) hepatic artery (HA) flow rate (mL/min/100 g liver), and (**d**) HA resistance (mmHg/min/100 g liver) values (mean ± standard deviation) during isolated liver reperfusion. There is no significant difference in hemodynamic data between HA and PV. However, HA resistance gradually increases in the CS group (*n* = 3), whereas it gradually decreases in the HMP group (*n* = 3). Statistical significance was set at *p* < 0.05. CS, cold storage; HMP, hypothermic machine perfusion.

During ILR, there was no significant difference in hemodynamic data between the HA and PV. However, HA resistance gradually increased in the CS group, whereas it gradually decreased in the HMP group. At the end of ILR, HA resistance in the HMP and CS groups was 0.106 ± 0.032 and 0.416 ± 0.191 mmHg/mL/min/100 g liver, respectively (*p* = 0.1) (Figure 4b,d).

### 3.3. Morphological Findings

**Figure 5:**
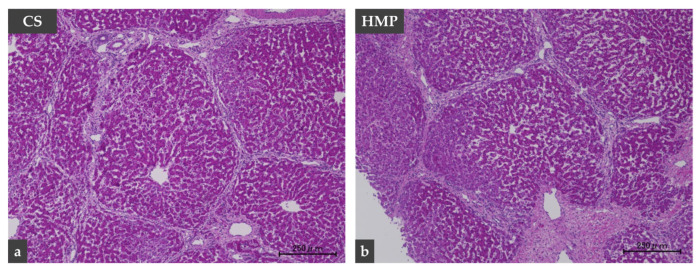
Histopathological findings. Hematoxylin and eosin staining show that the structure of liver cells is relatively well maintained in the CS (*n* = 3) and HMP (*n* = 3) groups. CS (**a**), cold storage; HMP (**b**), hypothermic machine perfusion.

Hematoxylin and eosin staining showed that the structure of liver cells was relatively well maintained in the CS and HMP groups (Figure 5). Sinusoidal congestion, hepatocyte cytoplasmic vacuolization, and parenchymal necrosis in the two groups appeared to be similar.

**Figure 6:**
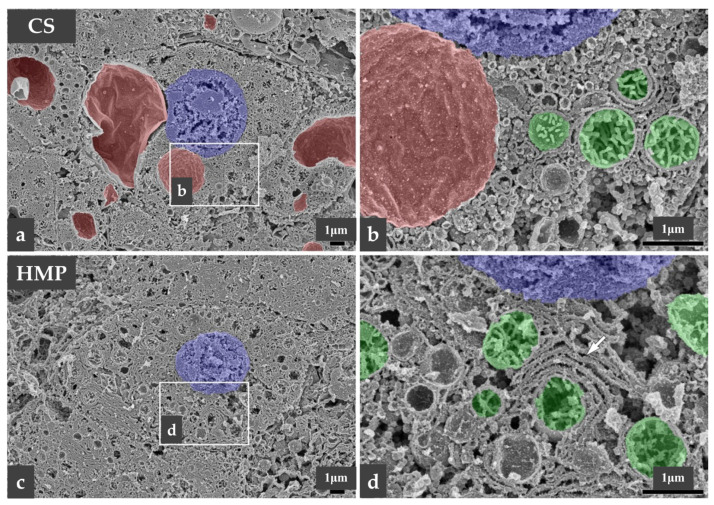
Electron microscopic findings. Low-magnification scanning electron microscopy showing vacuolar formation (**a**,**c**, colored red) in the liver samples from the CS group (*n* = 3), which is scarcely detected in the HMP group (*n* = 3). Under higher magnification, the mitochondria (**b**,**d**, colored green) in the cytoplasm of hepatocytes have a small and round shape, indicating a sufficient function in both CS and HMP samples. However, hepatocytes in the HMP group often have a flattened and lamellar endoplasmic reticulum (**d**, arrow). CS, cold storage; HMP, hypothermic machine perfusion.

In contrast, observation under low magnification (5000×) using osmium-maceration SEM (OM-SEM) showed vacuolar formation (Figure 6a,c) in the liver samples from the CS group, which was scarcely detected in those from the HMP group. These vacuoles corresponded to the liver damage, as reflected in the laboratory test results of AST, ALT, LDH, and ALP levels described in the previous section. Under higher magnification (20,000×), the mitochondria (Figure 6b,d) in the cytoplasm of hepatocytes had a small and round shape, indicating a sufficient function in both CS and HMP samples. However, the hepatocytes in the HMP group often had a flattened and lamellar endoplasmic reticulum (Figure 6d, arrow), indicating the production of necessary proteins.

## 4. Discussion

DCD has become the fastest-growing proportion of the donor pool. After the successful use of kidney grafts from DCD for clinical transplantation, interest has shifted toward using extrarenal organs such as the liver, pancreas, and lungs. However, in the early phase, liver transplantation from DCD does not always result in favorable posttransplant outcomes. In recent years, a new technology for preservation has been required, particularly for liver grafts from DCD. The use of ECMO perfusion is based on experimental studies that showed that the recirculation of oxygenated blood at 37 °C could improve the cellular energy load and reduce tissue injury to the liver during the period of warm ischemia induced by cardiac arrest [18,19]. After cardiac arrest, abdominal organs are reperfused and oxygenated while the potential DCD is evaluated and consent for organ donation is obtained. Fondevila et al. reported the remarkable effects of the combined use of in situ normothermic ECMO (NECMO) and normothermic MP (NMP) in donors 90 min after cardiac death in a porcine liver transplantation model. The advantage of NMP, including the use of NECMO, is its ability to overcome the disadvantages of hypothermic cellular physiology. NECMO initiates the processes of energy repletion and cellular repair, whereas the subsequent NMP provides the physiological conditions and substrates necessary for continued improvement in the grafts during ex vivo preservation [20,21].

However, normothermic perfusion requires high levels of oxygen, which means a need for an oxygen carrier such as hemoglobin. Blood-based perfusates may increase the risk of microvascular failure, sinusoidal plugging, and bacterial growth. Continuous anticoagulation administration is essential: unfractionated heparin is generally used. Some new anticoagulants including bivalirudin and nafamostat mesilate have been introduced in the context of ECMO. However, the feasibility of using these anticoagulants remains unclear; future research should be conducted to study their effects on preserved organs [22,23]. Therefore, achieving normothermic perfusion in the liver remains challenging. Subnormothermic perfusion performed at 20–21 °C resulted in reduced metabolic requirements, as well as lower vasoconstriction in steatotic and DCD rat models [24,25]. Tolboom et al. reported that subnormothermic MP at 20 °C or 30 °C reduced AST and ALT levels in rat livers after 1 h of warm ischemia [26]. Previously, we reported that the combined use of in situ subnormothermic ECMO and HMP-based preservation was more beneficial to the functional improvement in porcine liver grafts from DCD [27]. In this study, we compared CS and HMP in terms of the effects of the combined use of in situ subnormothermic ECMO. ECMO after an extended period of warm ischemia and subsequent HMP alleviated hepatocellular injury, as shown in Figure 3, although the difference was not significant.

We used both histopathology and electron microscopy for the morphological evaluation. Ultrastructural analysis with electron microscopy is widely used to assess tissue damage. Previous studies have shown that OM-SEM was useful in evaluating liver tissue damage. OM-SEM can be used to assess the detailed 3-D ultrastructure of a single cell, particularly in large, highly productive cells, such as hepatocytes [28,29]. In our study, OM-SEM findings showed that cytoplasmic vacuolization of hepatocytes was more frequently detected in the CS group than in the HMP group. Cytoplasmic vacuolization of hepatocytes is one of the changes that occur in ischemia–reperfusion injury (IRI) and is included in the Suzuki classification scored (from 0 to 4) for sinusoidal congestion, hepatocyte cytoplasmic vacuolization, and parenchymal necrosis for assessing IRI [30]. It has been suggested that HMP could reduce the incidence and severity of IRI and improve outcomes after organ transplantation [8,31]. Clinical organ retrieval requires a period of cold preservation during the transportation from the donor’s hospital to the recipient’s one. Compared to CS, HMP allows a continuous minimum level of physiological metabolism and avoids cold ischemic damage, which could recover from IRI. The preventive effect of IRI on HMP has also been demonstrated based on hematoxylin and eosin staining observations, with significantly better findings regarding hepatocyte cytoplasmic vacuolization and parenchymal necrosis in the HMP group than in the CS group [32]. In this study, although no clear differences were detected through hematoxylin and eosin staining observations, the protective effects against IRI induced by ECMO might have contributed to the reduction in such injury in both groups.

Bochimoto et al. previously revealed that subnormothermic MP at 22 °C preserved the functional ultrastructure of the mitochondria and rough endoplasmic reticulum (rER) well in hepatocytes [28,29,33], as well as reduced hepatocellular enzyme release [14]. This study also showed that these intracellular membrane structures in hepatocytes could maintain functional form even after ILR. In addition, oxygenation caused the lamellarization of the rER in hepatocytes after reperfusion, which appeared to be more effective. Our OM-SEM findings showed that hepatocytes in the HMP group often had a flattened and lamellar endoplasmic reticulum, suggesting that the combined use of subnormothermic ECMO and HMP may have more protective effects. Moreover, our OM-SEM findings showed the small and round shape of the mitochondria in the cytoplasm of hepatocytes in both groups. Mitochondrial-derived oxidative stress has been revealed as a key mechanism of tissue damage in the field of IRI [34,35,36]. Schlegel et al. demonstrated that hypothermic oxygenated perfusion (HOPE) for ischemic liver could prevent mitochondrial impairment and protect against subsequent IRI. The mechanism of mitochondrial-based liver IRI is initiated by succinate accumulation and flavin mononucleotide (FMN) release from the mitochondria during liver reperfusion, which leads to a multifactorial injury including mitochondrial reactive oxygen species release, danger-associated molecular pattern signaling, and activation of non-parenchymal liver cells. The release of mitochondrial FMN depends on mitochondrial succinate and is significantly reduced under HOPE. HOPE induces a metabolic switch in mitochondria through an improved enzymatic activity of complexes I, II, and IV. After HOPE, such findings correlate with a decrease in lactate and succinate in liver tissues, an increase in the mitochondrial adenosine triphosphate level (ATP), and a reduced nicotine adenine dinucleotide (oxidized/reduced) ratio [37]. In this study, the preserved ultrastructure of mitochondria in the CS group suggests the possibility that ECMO has a protective effect on mitochondria. Another pilot study reported that the FMN value at 30 min of NECMO may help clinicians to decide to accept DCD donors or not [38,39], and further research on this is needed in the future.

This study has several limitations. First, we used an experimental pig model, and an actual transplantation was not performed. Furthermore, the effect was limited by the short observation period. The ILR model is commonly used as a valuable model to study events of the early reperfusion phase of the ischemia reperfusion cascade that occurs during organ transplantation [40]. However, even when observed for an extended period of time, it significantly differs from the actual IRI during transplantation due to using diluted autologous blood as the reperfusion fluid. Therefore, we considered that a 2 h assessment after reperfusion would be adequate. In addition, bile assessment could not be performed in this study, primarily due to obtaining unstable samples. We suggest that the use of diluted autologous blood may have contributed to this issue. However, the relatively short observation period may also have played a role. Hence, actual transplantations and long-term observations need to be conducted. Second, we could not show significant differences in several analyzed parameters due to the small sample size. However, we believe that this study is promising, as it presents data obtained from a large animal model. Third, we did not measure any biochemical parameters that could reflect the mitochondrial functionality, such as ATP and FMN levels. Their measurement may help to evaluate the vitality of the mitochondria in detail. Finally, we only observed hepatocytes using electron microscopy. Transmission electron microscopy (TEM) is a powerful and convenient tool for morphological studies of small cells such as liver sinusoidal endothelial cells. Therefore, TEM should be considered in future studies.

## 5. Conclusions

The combined use of in situ subnormothermic ECMO and HMP was more beneficial for the functional improvement in liver grafts from DCD. Further studies are needed to determine the extent of clinical application of this combined technology.

## Figures and Tables

**Table 1:** Composition of preservation solution.

Medication Name	Brand Name and Manufacturer	Quantity	
Histidine-tryptophan-ketoglutarate solution	Custodiol; Dr. Franz Köhler Chemie GMBH, Bensheim, Germany	500	mL
5% albumin	Kenketu Albumin 25% for I.V. Injection 12.5 g/50 mL; Takeda Pharmaceutical, Tokyo, Japan	250	mL
Vitamin	Vitaject Injection Kit; Terumo, Tokyo, Japan	10	mL
Amino acid	Hikarilevan; Nihon Pharmaceutical, Tokyo, Japan	200	mL
Glutathione	Tathion for Injection; Choseido Pharmaceutical, Tokushima, Japan	1000	mg
Fast-acting insulin human	Novolin R Injection; Novo Nordisk Pharma, Tokyo, Japan	50	units
Methylprednisolone sodium succinate	Solu-Medrol for Intravenous Use; Pfizer, Tokyo, Japan	250	mg
8.4% sodium bicarbonate	Meylon Injection 8.4%; Otsuka Pharmaceutical Factory, Naruto, Japan	20	mL

## Data Availability

The datasets generated and/or analyzed in the current study are available from the corresponding author upon reasonable request.
